# Study on the Shear Resistance Performance of Grouped Stud Connectors

**DOI:** 10.3390/ma16206625

**Published:** 2023-10-10

**Authors:** Wenru Lu, Yuanming Huang, Wenhan Xu

**Affiliations:** 1 School of Civil Engineering, Henan University of Technology, Zhengzhou 450001, China; h15515633883@163.com; 2 Henan Key Laboratory of Grain and Oil Storage Facility & Safety HAUT, Zhengzhou 450001, China; 3 Henan Urban Planning and Design Institute Co., Ltd., Zhengzhou 450044, China; 4 Zhengzhou Research Institute, Harbin Institute of Technology, Zhengzhou 450000, China

**Keywords:** grouped stud connectors, finite element analysis, grouped stud effect, calculation of shear capacity

## Abstract

In order to further investigate the grouped stud effect on the force properties of stud connectors, based on the premise that the correctness of the finite element simulation method, in this paper, a finite element model of grouped stud connectors was developed, and the grouped stud effect and its sensitivity factors were analyzed in order to validate the recommended formula for calculating the shear capacity of grouped stud connectors. Results show that the number of grouped stud rows and stud row spacing have a significant influence on the grouped stud effect, and the unevenness coefficient of grouped stud force is negatively correlated with the number of grouped stud rows as well as the grouped stud row spacing. Grouped stud connectors with commonly used concrete grades greater than C50 and height-to-diameter ratios of greater than 4 in steel–concrete composite structural bridges are insensitive to changes in the concrete strength grades and the length of the studs. The direction of force transmission for grouped stud changes with the change in loading angle and the unevenness coefficient of force for the grouped stud will therefore be reduced. By comparing the results of the 62 existing groups of grouped stud connectors push-out tests, the mean of the tested to calculated value ratio was found to be 1.12, the variance was 0.023, the dispersion was small, and it was shown that the recommended formula has a high degree of accuracy. The results of this paper can be used as a theoretical basis for the study of the shear stress performance of grouped stud connectors.

## 1. Introduction

As a key synergistic force-transferring member of steel–concrete composite structures, stud connectors are now widely used in steel–concrete composite beams, hybrid beams, composite columns, and other structures [1,2,3]. The shear force between the steel member and concrete member is mainly borne by the connection, while resisting the role of lift-off, which is the key stress member of the steel–concrete composite structure [4,5,6]. Grouped stud connectors have the advantages of isotropy, high shear bearing capacity, good lifting resistance, easy construction, and so on, and are thus the most widely used shear connectors [7,8,9,10,11,12].

The shear performance of stud connectors have been of interest to scholars for decades, a large number of studies have shown that the main forms of damage of stud connectors under shear include the shear damage of studs and the compression damage of concrete [13]. The shear resistance of the studs is mainly affected by the stud material, stud body diameter, stud length, weld quality between the stud and steel plate, concrete strength, and hoop ratio [6,14,15,16]. The method of calculating the shear capacity of a single stud was creatively proposed by Viest and is widely used in several national codes [17,18,19,20].

Wang et al. [21] analyzed the sensitivity of parameters such as stud diameter and concrete strength with the help of finite element modeling, and a new formula for calculating the shear capacity of stud connectors was proposed. More and more novel stud connectors have been invented by research scholars and shear calculation methods were proposed, which are only applicable to the novel connectors [22,23,24] and are not generalizable.

With the continuous improvement of the road grade and the expansion of the construction scale, the application of cluster grouped stud connectors in steel–concrete composite structures is more widely used. For dense, large-sized grouped stud connectors under loads, the distribution of shear force across the studs was heterogeneous [25], the average shear capacity and shear stiffness of single stud of grouped stud connectors were significantly lower than those of single stud specimens [26,27,28]. With the increasing number of grouped stud rows in the direction of force, the degree of heterogeneity of the grouped stud shear would increase [26,28]. Through a parametric sensitivity analysis of the grouped stud connectors, it was found that grouped stud spacing, number of grouped stud rows in the loaded direction, concrete and stud material properties, and stud size are the main factors affecting the shear performance of grouped stud connectors [23,27,29]. For fiber-reinforced concrete grouped stud connectors, because the presence of fibers affects the ductility of the studs, the stress concentration at the interface between the studs and the concrete will be reduced [6,30]. Much scholarly attention has also been paid to the force properties of grouped studs in assembled composite beams [10,21,31,32,33]. Most current studies have concluded that the model provided in the Chinese design code for calculating shear capacity is conservative, and the design provisions for stud shear connectors are given in the Eurocodes, but none of the formulas take into account the contribution of the concrete strength to the connection: when the concrete strength class is above a certain value, its shear capacity no longer changes [17]. On the basis of the analysis, new formulas or adjustment factors for calculating the shear capacity of grouped stud connectors in design codes have been proposed [34,35,36,37,38]. In summary, the shear performance of single-row stud connectors has been analyzed by the previous literature; however, limited research has been conducted on the force behavior of grouped stud connectors and systematic analyses of the stress performance of grouped stud connectors are also rare.

The refined finite element analysis method was used to simulate the push-out test of existing single-row stud connectors in this paper, based on the premise that the correctness of the finite element simulation method, multi-row stud finite element models were developed. In order to investigate the influence of grouped stud effect on the shear resistance performance of grouped stud connectors. By numerically analyzing the grouped stud effect and the main factors affecting it, the grouped stud effect of grouped stud connectors and the main factors affecting the grouped stud effect were numerically analyzed. On the basis of test data from the introduction of a total of 62 grouped stud connectors at home and abroad, the formula for calculating the shear capacity of grouped stud connectors is recommended.

## 2. Finite Element Simulation and Verification

**Figure 1:**
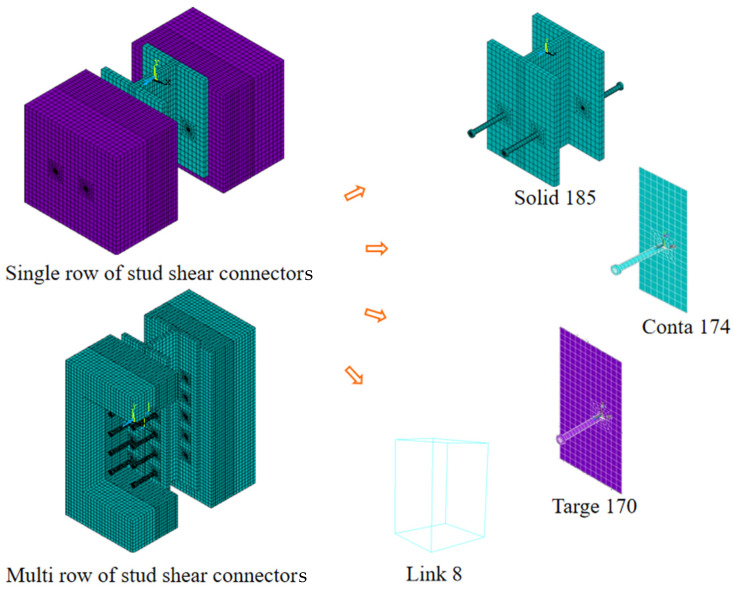
Finite element model of stud shear connectors.

Considering steel and concrete material nonlinearities, to perform refined finite element simulations of the stud shear connectors push-out test pieces, the large-scale general-purpose finite element analysis software ANSYS was employed (ANSYS19.2, Southpointe 2600 Ansys Drive, Canonsburg, PA, USA). The finite element model is schematically shown in Figure 1. The stud shear connectors are composed of two rows of φ22 × 200 studs with spacings of 200 mm horizontally on each side, the concrete material is C50, the stud material is ML15, and the steel plate material is Q345. The stud and steel plate were simulated using Solid185, a solid unit; the concrete was simulated using the tetrahedral solid unit Solid65; ordinary steel bars were simulated using the bar unit Link8 to achieve the synergistic deformation of plain steel and concrete; and three-way translational displacement degrees of freedom between ordinary steel nodes and corresponding concrete nodes were constrained. Both the steel plate–concrete block and the stud–concrete block were simulated using the face-to-face contact unit Conta174 and the target unit Targe170, respectively, and a friction coefficient of 0.4 was achieved [39]. The actual loading was simulated by applying a face load on the top surface of the steel plate, and the three-way translational displacement degrees of freedom of the bottom edge node of the concrete block were constrained [40].

**Figure 2:**
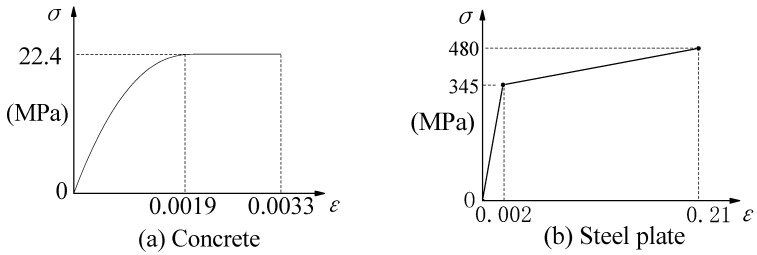

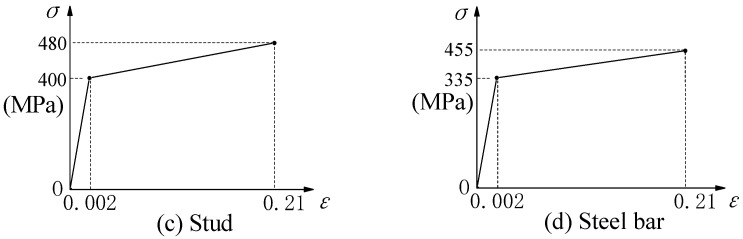
Constitutive relationship curves.

The materials in the model were defined using an elasto-plastic constitutive model, and their stress–strain relationship curves are shown in Figure 2.

**Figure 3:**
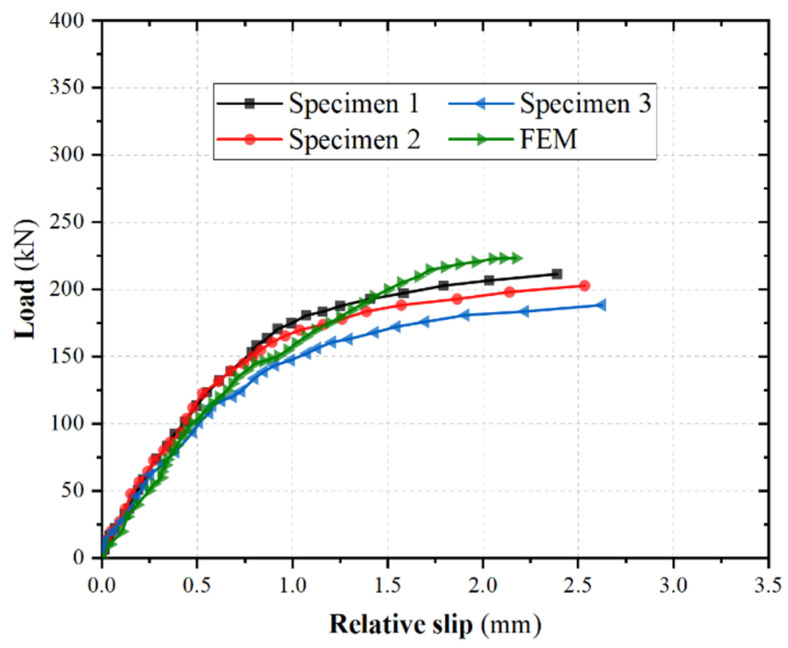
Load–slip curves.

In the literature [39], a push-out test was carried out on the single row of stud connectors in Figure 1, the specimen eventually underwent bending shear damage at the root of the studs, and the average shear bearing capacity of 230 kN for single stud was obtained. The calculated shear capacity is only 2.1% different from the test result; the calculated load–slip curve is shown in Figure 3. It can be seen that the load–slip curves of the finite element model (FEM) calculation results and the test results in the elastic–plastic phase are in good agreement, indicating that the actual stress condition of the shear stud specimen in this paper is more accurately simulated via the finite element method. According to methods found in the literature [41], the slope of the cut line at the 1/3 size of the shear capacity of the stud shear connection is taken as the shear stiffness of the connector, the average shear stiffness of the single stud was 206 kN·mm^−1^. The results of finite element analysis of this single row of stud shear connectors in this paper show that the average shear capacity and shear stiffness of single nails were 223 kN and 217 kN·mm^−1^, respectively. The difference between simulated and experimental measured results is 3.0 and 5.4%, and the reliability and computational accuracy of the finite element analysis method is thus confirmed in this paper.

## 3. Properties and Damage Mechanism

### 3.1. Critical Stud Spacing for the Grouped Stud Effect

**Figure 4:**
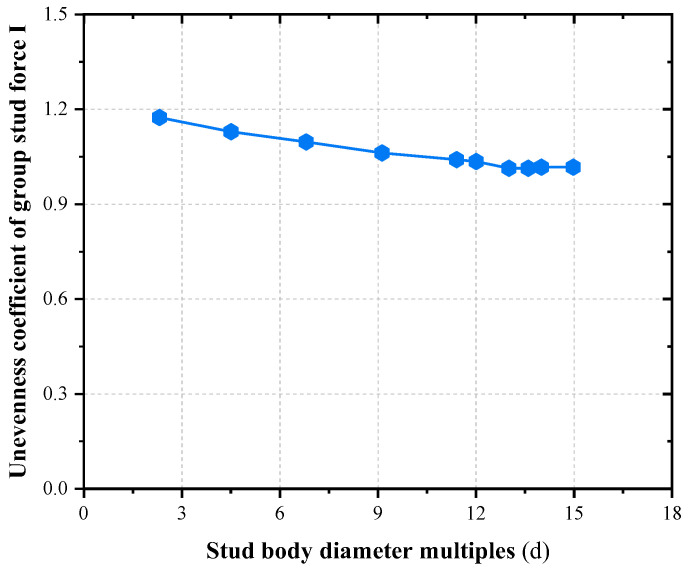
Stud body diameter multiples and unevenness coefficient of grouped stud force.

Stud connectors are often employed in composite structures in the form of grouped stud. It is possible to follow the single stud design in some cases, but not in others, it depends on the magnitude of the concrete stresses in the surrounding studs [42,43]. Within a certain range, its grouped stud effect diminishes with increasing grouped stud spacing, increases with increasing the shear force, and increases with increasing overlap of concrete pressure cracking zones [44,45,46]. Generally, the grouped stud effect is more significant in shallow concrete than in deeper concrete; the grouped stud effect in the direction of load action is greater than that in the direction of perpendicular load action. The reduction in the average shear capacity of a single stud is the most intuitive manifestation of the grouped stud effect and enables the characterization of the degree of reduction in the average shear capacity of a single stud due to the grouped stud effect. Where the grouped stud efficiency is defined by the unevenness of force coefficient *I*, coefficient *I* is shown in Formula (1). Different stud row spacings were analyzed by the authors, and the results are shown in Figure 4; when the grouped stud row spacing in the direction of load application is up to 13 times the stud diameter, the shear value of the grouped stud tends to be homogeneous and the effect of group stud effect on the shear force of the stud group can be neglected. At this point, its grouped stud efficiency is close to 1.0, and it can be considered a single stud, and when it is less than 13 d (d is the stud body diameter), the grouped stud effect should be taken into account, and the values are taken to be consistent with the code. The findings of this study are in line with the literature [47].

(1) $$I=\frac{\max.\left(F_{1},F_{2},\cdots ,F_{i}\right)}{\sum_{j=1}^{n}Q_{gu}/n}$$

where *I* is the unevenness coefficient of grouped stud force; *F*_i_ is the shear value of each stud in the grouped stud connectors; *Q_gu_* is the shear capacity of the grouped stud shear connectors; and *n* is the number of studs rows corresponding to the direction of the load.

### 3.2. Working Properties and Damage Mechanism of Grouped Stud and Influencing Factors

#### 3.2.1. Effect of Grouped Stud Row Spacing in the Direction of Shear Action

**Figure 5:**
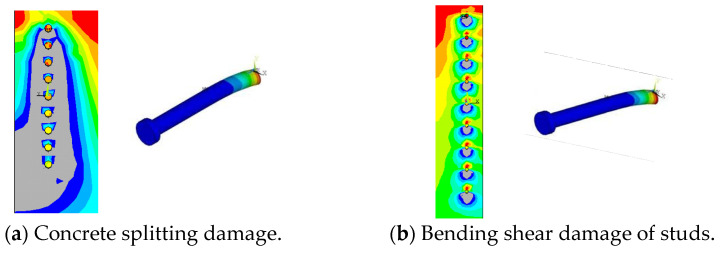
Connectors damage pattern.

**Figure 6:**
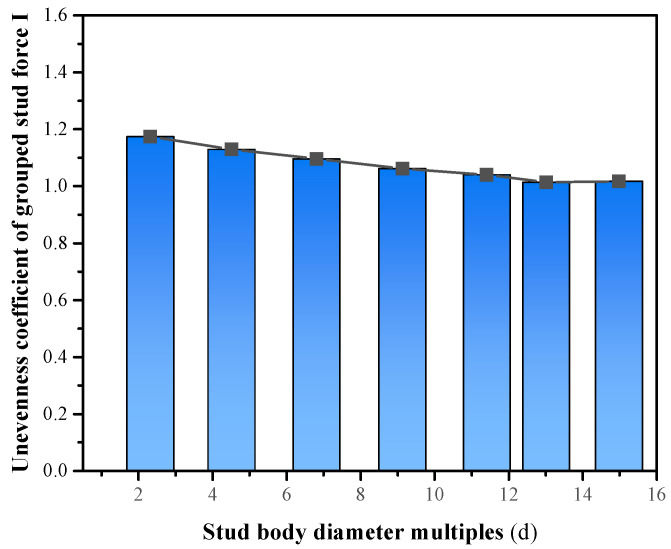
Stud body diameter multiples and unevenness coefficient of force.

**Figure 7:**
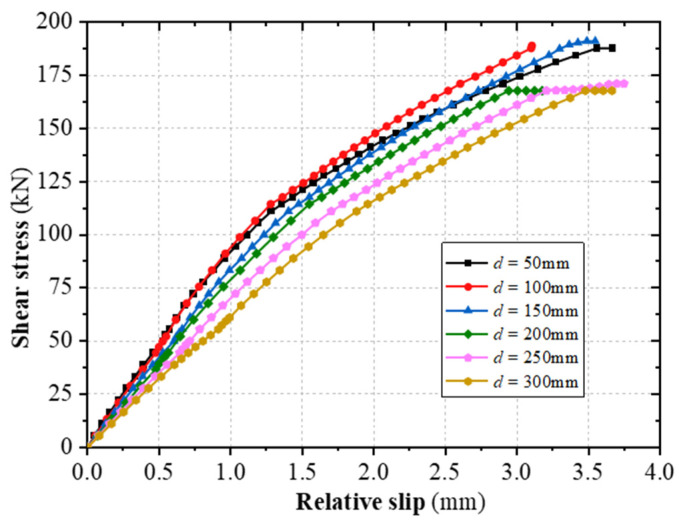
Effect of stud row spacing on load–slip curves.

The spacing (d) between stud rows was set to 50 mm (2.3 d), 100 mm (4.5 d), 150 mm (6.8 d), 200 mm (9.1 d), 250 mm (11.4 d), and 300 mm (13.6 d), respectively, the effect of row spacing on the force properties of grouped stud connectors was analyzed. The results show that splitting damage of the concrete occurred in the connector (Figure 5a) when the grouped stud spacing was 50 mm, bending shear damage of the studs occurred in the remaining specimens (Figure 5b). This was caused by the interaction of concrete between grouped stud when the grouped stud is subjected to shear forces, overlapping stress distributions occur in the concrete between the grouped stud, and its overlap effect decreases with increasing stud spacing, and the unevenness of the grouped stud force decreases (Figure 6 and Figure 7). Consequently, when the stud spacing is small, the distribution of concrete stresses has a significant superposition effect, the connectors may even undergo damage in the form of splitting damage of the concrete (e.g., d = 50 mm); when the stud row spacing is large, the distribution of concrete stresses has less overlapping effect, and when the stud row spacing is more than 13 times the stud body diameter, the unevenness of the grouped stud force is close to 1, i.e., close to the same state of force as that of a single stud.

#### 3.2.2. Effect of the Grouped Stud Rows

**Figure 8:**
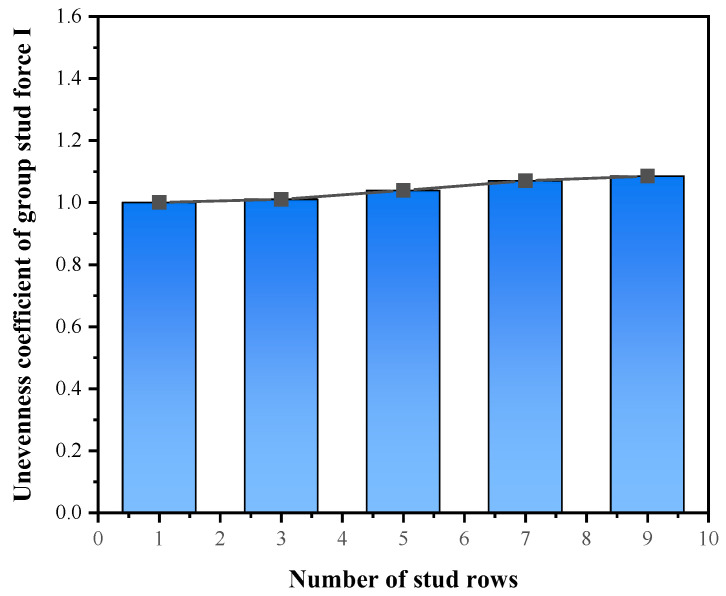
Number of stud rows and unevenness coefficient of force.

**Figure 9:**
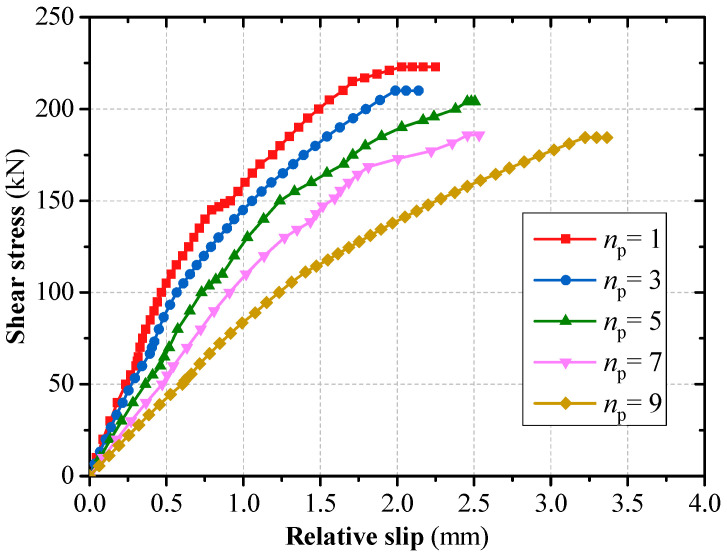
Effect of the number of stud rows on the load–slip curves.

Keeping the grouped stud row spacing at 150 mm, the numbers of grouped stud rows *n_p_* were set as 1, 3, 5, 7, and 9, and the effect of row spacing on the force properties of grouped stud connectors was analyzed. The results show that the bending shear damage of the studs occurred in all of these specimens; the unevenness coefficient of grouped stud force increases with the increase in the number of grouped stud rows (Figure 8); and the shear capacity of the connectors decrease with the increasing number of grouped stud rows, and the decreasing trend is significant. When the number of rows is increased from 1 to 9, the average shear capacity of single stud is reduced by 17.5% (Figure 9).

#### 3.2.3. Effect of Concrete Strength Grade

**Figure 10:**
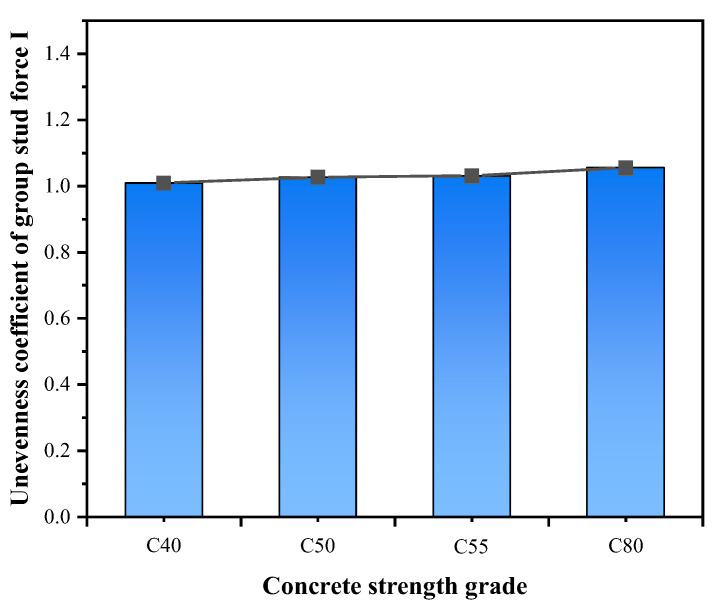
Concrete strength grade and unevenness coefficient of force.

**Figure 11:**
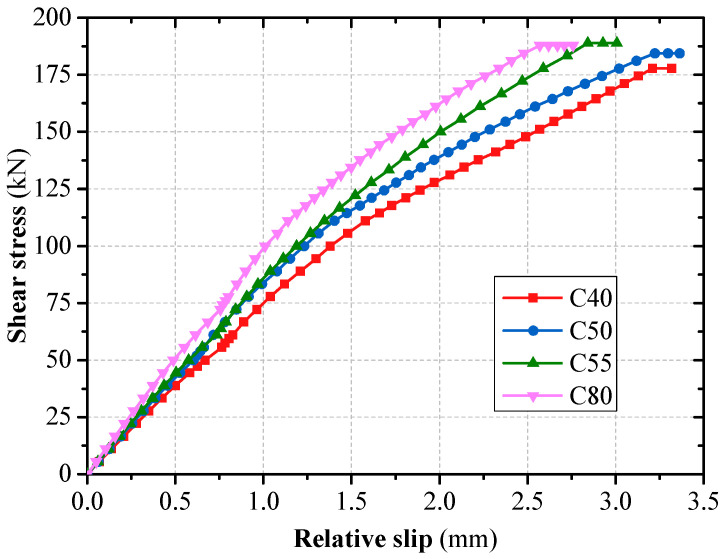
Concrete strength grade on the load–slip curves.

The deformation and force of the grouped stud will be restrained by the concrete, however, when the concrete strength exceeds C50, the shear capacity increase of the shear connectors is relatively reduced, the unevenness coefficient of grouped stud force also varied less (Figure 10 and Figure 11). When the concrete strength grade is increased from C50 to C80, the average shear capacity of single stud is increased by only 0.53%, the unevenness coefficient of grouped stud force increased by only 2.36%, which is also consistent with the findings of the literature [10]. In combined structure bridges, concrete strength grade is normally C50 or higher. Therefore, the effect of concrete strength grade could be disregarded in the design of grouped stud for combination structure bridges.

#### 3.2.4. Effect of Stud Body Length

**Figure 12:**
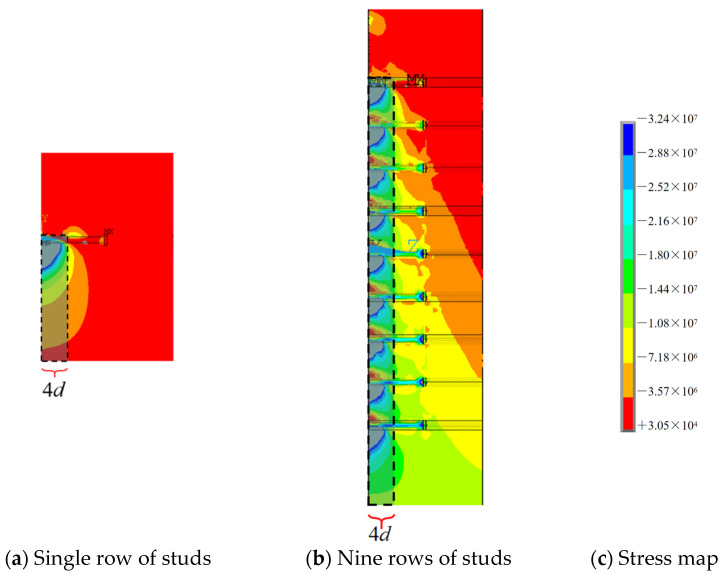
Concrete stress distribution in the length direction of the stud.

The stresses in the concrete are caused by the deformation of the studs in grouped stud connectors. The difference of stresses in the concrete around the weld end and the large head end of each stud is more pronounced, this is due to the crossover of the areas of concrete stress change caused by the deformation of the studs. For grouped stud connectors under vertical shear, the more significant overlap of the concrete stress distribution is essentially located at four times the depth of the stud diameter inside the concrete (Figure 12), i.e., where the studs are more shallowly embedded. Chinese steel structure code states that when the ratio of the height and diameter of grouped stud is more than 4, the increase of the height to diameter ratio has less effect on the shear capacity of grouped stud [48]. Studs used in bridges are required to have a height-to-diameter ratio greater than 4, thus, the shear capacity of grouped stud is generally ignored in the calculation.

#### 3.2.5. Effect of Force Angle

**Figure 13:**
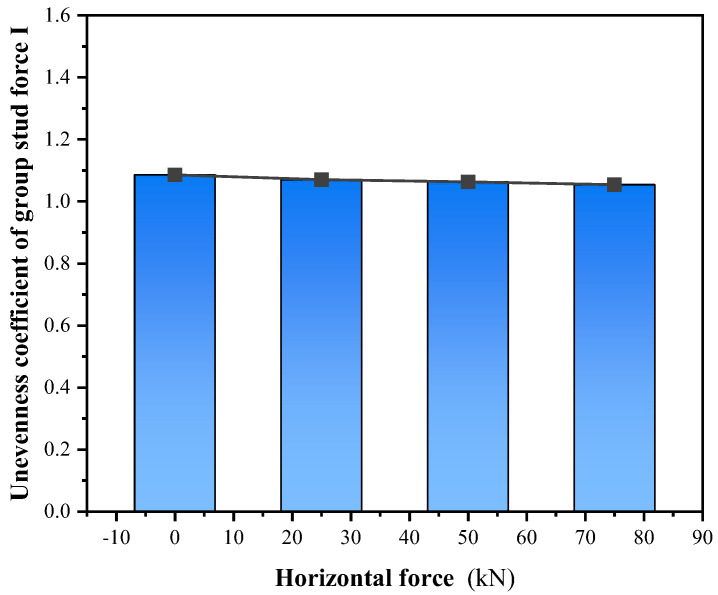
Force angle and unevenness coefficient of force.

**Figure 14:**
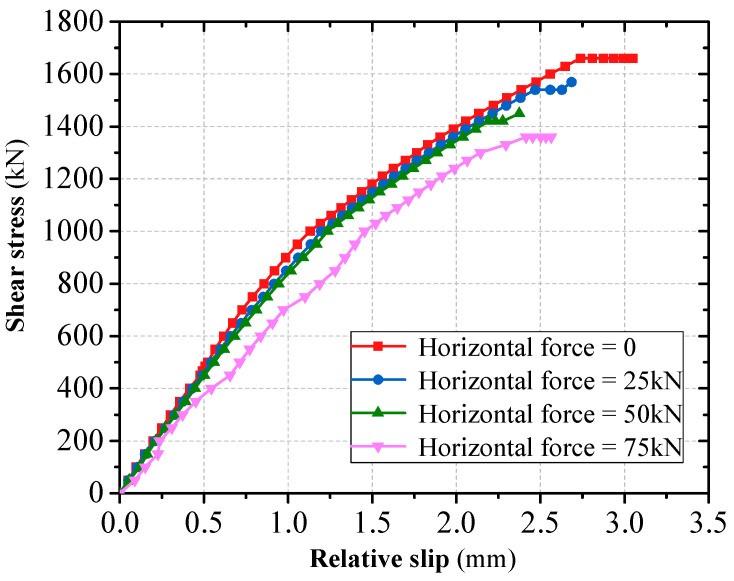
Effect of force angle on load–slip curves.

**Figure 15:**
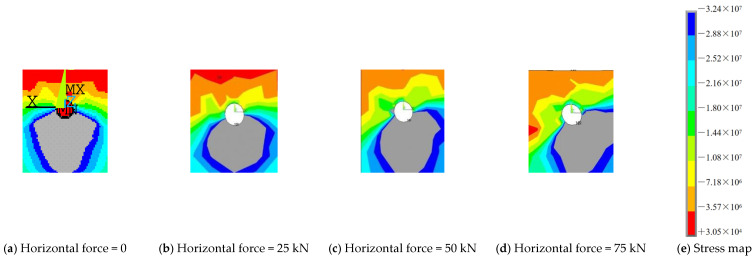
Concrete main compressive stress diagram for damage load of multi-row stud connectors.

Through considering the vertical shear and diagonal shear, the shear resistance of grouped stud connectors was analyzed. When simulations in finite elements for oblique shear were performed, the simulations were performed using a certain horizontal force applied simultaneously with a varying vertical force. When the calculated horizontal force is 0 kN, 25 kN, 50 kN, and 75 kN, respectively, the results of the ultimate vertical load capacity of the connection are shown in Figure 13 and Figure 14. The body of the single stud is round in cross-section, and its forces are not affected by the loading angle of the load. However, for grouped stud connectors, when the loading angle is changed, the direction of force transmission of the grouped stud is altered (Figure 15). When conducting the force analysis of the grouped stud, the load can be decomposed into components that are aligned with the direction of the stud row and column according to the arrangement of the stud row and column. It is recommended that during design, the rows of grouped stud are arranged in the loading direction to facilitate the analysis of the forces on the grouped stud.

## 4. Shear Capacity of Grouped Stud Connectors

According to the existing theories and data results, the formula for calculating the shear capacity *Q_gu_* of grouped stud connectors is recommended, as shown in Formulas (2) and (3) [36].

(2) $$\{\begin{array}{cc} \varphi =1 & n_{p}\leq 3\\ \varphi =0.9392+0.0423n_{p}-0.0079n_{p}{}^{2} & 3<n_{p}\leq 5\\ \varphi =0.9776-0.0034n_{p}-0.0003n_{p}{}^{2} & 5<n_{p}\leq 15 \end{array}$$

(3) $$\left\{ \begin{array}{ll} Q_{gu}=\varphi \cdot n_{p}\left(0.5A_{s}\sqrt{E_{c}f_{ck}}\right) & A_{s}\sqrt{E_{c}f_{ck}}\leq 344000\\ Q_{gu}=\varphi \cdot n_{p}\left(0.26A_{s}\sqrt{E_{c}f_{ck}}+165A_{s}\right) & A_{s}\sqrt{E_{c}f_{ck}}>344000 \end{array}\right.$$

where *φ* is the average shear capacity reduction coefficient of the single stud of the grouped stud shear connectors; *A_s_* is the cross-sectional area of the stud body (mm); *E_c_* is the modulus of elasticity of concrete (MPa); and *f_ck_* is the axial compressive strength of concrete (MPa).

**Figure 16:**
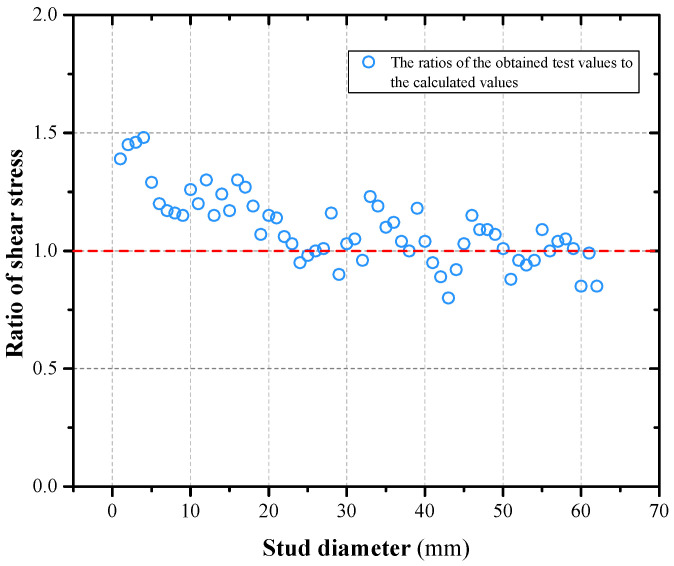
Comparison between the calculation formula of grouped stud and the actual push-out specimens.

Push-out tests on the shear performance of grouped stud connectors have been carried out both at home and abroad, and the test values of shear capacity for different number of studs and different specimen sizes have been obtained [40,49,50,51,52,53,54,55,56,57,58,59,60,61,62,63,64,65,66,67,68,69,70,71,72]. The design parameters of 62 specimens were referenced in this paper, the calculated values of shear capacity of grouped stud connectors were calculated according to Formulas (2) and (3), the ratios of the obtained test values to the calculated values are summarized in Figure 16. The average value of the ratio of the test and calculated shear capacity values of the grouped stud connectors is found to be 1.12 and the variance is found to be 0.023. It can be concluded that the dispersion of the calculated results of this method from the experimental values is small, which is in good agreement with the measured values, and it can be used as a reference for shear force analysis of related grouped stud connectors.

## 5. Conclusions

To study the shear performance of the grouped stud connectors, based on the push-out test, a refined finite element analysis method for grouped stud connectors has been developed, the grouped stud effect of the connectors and the main factors affecting the grouped stud effect have been numerically analyzed, and the formulae for calculating the shear capacity of grouped stud connectors are recommended. The main conclusions obtained are as follows:

(1) The shear performance of grouped stud connectors is affected by the grouped stud effect; this is related to the overlapping effect of the concrete compression cracking zones between the studs. When stud groups are densely arranged, the reduction in the average shear capacity of the single stud of the connector needs to be considered. When the stud spacing in the direction of load is less than 13 times the diameter of the stud body, the influence of the grouped stud effect needs to be taken into account in the design, considering the shear force reduction; otherwise, it is calculated as a single stud design.
(2) The inhomogeneity of the shear distribution of the stud group under load is presented, which is the most intuitive presentation of the grouped stud effect. At the same time, the average shear capacity of individual stud decreases as the shear inhomogeneity of the stud group increases. As the stud row spacing in the direction of load application becomes smaller or the number of rows increases, the more significant the superposition effect of the distribution of concrete stresses in the connectors, and the phenomenon of non-uniformity in the shear distribution of the stud group is also more significant. Grouped stud connectors with commonly used concrete grades greater than C50 and height-to-diameter ratios greater than 4 in steel–concrete composite structural bridges are insensitive to changes in the concrete strength grades and the length of the studs.
(3) The body of the single stud is round in the cross-section, and its forces are not affected by the loading angle of the load. However, for grouped stud connectors, when the loading angle is changed, the direction of force transmission of the grouped stud is altered. When conducting the force analysis of the stud group, the load can be decomposed into components that are aligned with the direction of the stud row and column according to the arrangement of the stud row and column. It is recommended that during design, the rows of grouped stud are arranged in the loading direction to facilitate the analysis of the forces on the grouped stud.
(4) The calculation of group stud connectors is not mentioned in the Chinese code. The recommended formula for calculating the shear capacity of group stud connectors is given in this paper, and the verification analysis was carried out by combining the results of 62 groups of existing grouped stud connector push-out tests. Results show that the mean value of the ratio of tested to calculated values is found to be 1.12, the variance is 0.023, the dispersion is small, the recommended formulas have high accuracy. The results of this paper are provided as a theoretical basis for the study of the shear performance of grouped stud connectors.
(5) At present, to meet the needs of low-carbon as well as assembled bridge structures, a large number of steel–concrete combined bridge structural connectors with new materials and new structural forms have appeared. In view of this, we will devote ourselves to the research on the stress performance of combined structural shear connectors to provide a solid theoretical foundation for the popularization and application of the connectors.

## Data Availability

All data generated or analyzed during this study are included in this article. All data included in this study are available upon request by contact with the corresponding author.
